# Efficacy of Magnetic Resonance Contrast-Enhanced Vessel Wall Imaging as an Ancillary Examination for Subarachnoid Hemorrhage With Bleeding Points Difficult to Determine on Conventional Vascular Imaging: A Report of Three Cases

**DOI:** 10.7759/cureus.23313

**Published:** 2022-03-19

**Authors:** Yuta Koketsu, Takafumi Tanei, Takenori Kato, Toshinori Hasegawa

**Affiliations:** 1 Neurosurgery, Komaki City Hospital, Komaki, JPN

**Keywords:** magnetic resonance contrast-enhanced vessel wall imaging, dissection, small aneurysm, subarachnoid hemorrhage, vessel wall imaging

## Abstract

Here, we have demonstrated the efficacy of magnetic resonance contrast-enhanced vessel wall imaging (CE-VWI) as an ancillary examination for subarachnoid hemorrhage with bleeding points difficult to confirm by conventional vascular imaging. Case 1 had a ruptured small aneurysm with a size of 1.8 × 1.1 mm at the origin of left anterior choroidal artery. CE-VWI showed enhancement of the apex of the aneurysm. Surgical clipping was performed by a mini-clip. In Case 2, a ruptured small aneurysm, 2.1 × 1.9 mm, was detected at right middle cerebral artery bifurcation. CE-VWI showed enhancement of the aneurysmal wall. Endovascular coil embolization was performed. In Case 3, irregular dilatation of left internal carotid artery (ICA) was detected. CE-VWI demonstrated enhancement of the dilatation wall. The lesion was deemed to be a dissection of the ICA or a blood blister-like aneurysm. Endovascular treatment using intracranial stent placement was performed, and the patient has had no rebleeding events for one and a half years. In all cases, conventional vascular imaging detected scanty morphological changes, and CE-VWI information provided reliable confirmation of the lesions as bleeding points.

## Introduction

Magnetic resonance (MR) vessel wall imaging (VWI) is used for the evaluation of intracranial vascular lesions such as atherosclerosis, vasculitis, and aneurysms [1]. Recently, the utility of contrast-enhanced (CE) VWI for subarachnoid hemorrhage (SAH) with multiple or small aneurysms to identify bleeding points has been reported [2-6]. In most patients with spontaneous SAH, ruptured aneurysms are detected as the bleeding source on conventional morphological vascular imaging such as digital subtraction angiography (DSA) or computed tomography (CT) angiography. However, when the aneurysms are small or morphological changes are scanty, it is difficult to determine the bleeding point only by conventional vascular imaging [3]. In this report, the efficacy of CE-VWI as an ancillary examination for SAH where it was difficult to confirm the bleeding points by conventional vascular imaging is described.

Imaging protocol

CE-VWI was acquired on a MR scanner with a 32-channel head coil (Ingenia 3.0 Tesla; Philips Healthcare, Amsterdam, The Netherlands). The vessel wall protocol included time-of-flight MR angiography and a 3D T1-weighted black blood vessel wall sequence with improved motion-sensitized driven equilibrium (turbo spin echo acquisition with a field of view, 18 × 18 cm^2^; voxel size, 0.7 × 0.7 × 0.7 mm^3^; total slab thickness, 70 mm; repetition time/echo time 410/8 ms; refocusing flip angle, 90°) before and after intravenous administration of gadolinium (0.1 mmol/kg), followed by a 40-ml saline flush. The vessel wall sequence was performed using the axial images, whereas the sagittal and coronal images were created by computed reconstruction. Pre- and post-gadolinium subtracted T1-weighted black blood vessel images were also acquired.

## Case presentation

Case 1

**Figure 1:**
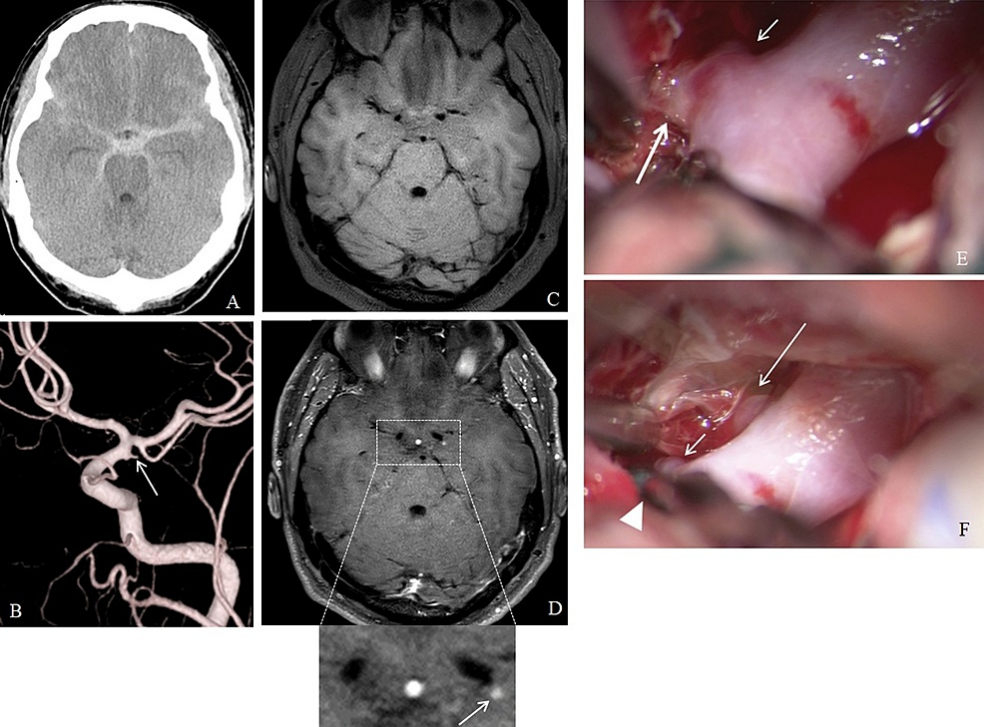
Case 1 (A) Computed tomography shows diffuse subarachnoid hemorrhage. (B) Digital subtraction angiography demonstrates a small aneurysm (arrow). Magnetic resonance vessel wall imagings (C) pre-contrast and (D) post-contrast administration show enhancement of the apex of the aneurysm (arrow). (E, F) Intraoperative photographs show the aneurysm surrounded by subarachnoid hemorrhage (thick arrow: aneurysm, short arrow: anterior choroidal artery, long arrow: posterior communicating artery, arrowhead: mini-clip).

A 46-year-old man presented with a sudden-onset severe headache. Computed tomography (CT) showed diffuse SAH (Figure 1A). Digital subtraction angiography (DSA) demonstrated a spindle-shaped bulge 1.8 × 1.1 mm at the origin of left anterior choroidal artery (AChA) of the left internal carotid artery (ICA) (Figure 1B). CE-VWI was performed on the day of onset, and the apex of the bulge was enhanced (Figures 1C, 1D). Therefore, the bulge was deemed a ruptured small aneurysm. On intraoperative findings, the small aneurysm was surrounded by SAH (Figure 1E). Hence, the aneurysm was considered to be the rupture point. Surgical clipping was performed by a mini-clip using intraoperative motor evoked potential monitoring (Figure 1F). The patency of AChA was confirmed by intraoperative surgical microscope-integrated indocyanine green video angiography. The patient was discharged without neurological deficits.

Case 2

**Figure 2:**
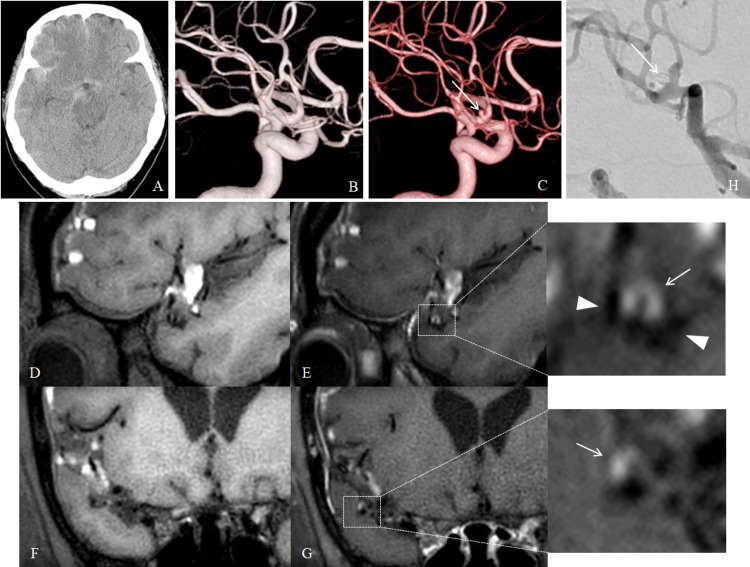
Case 2 (A) Computed tomography shows subarachnoid hemorrhage in the right Sylvian fissure. (B) Initial digital subtraction angiography did not detect any vascular lesions. (C) Second digital subtraction angiography shows a small aneurysm (arrow). (D, E) Sagittal and (F, G) coronal views of magnetic resonance vessel wall imagings (D, F) pre-contrast and (E, G) post-contrast administration show enhancement of the aneurysm wall (arrow: enhancement, arrow heads: middle cerebral arteries). (H) Digital subtraction angiography after endovascular coil embolization shows disappearance of the aneurysm (arrow).

A 48-year-old woman presented with a sudden-onset severe headache. CT showed SAH in the right Sylvian fissure (Figure 2A). DSA did not show any vascular lesion causing SAH (Figure 2B). Six days later, the second DSA showed a small, round bulge, 2.1 × 1.9 mm, at right middle cerebral artery (MCA) bifurcation (Figure 2C). The same day, CE-VWI showed enhancement of the bulge wall (Figures 2D-2G). The bulge was judged to be a small ruptured aneurysm, and endovascular embolization was performed using three coils (Figure 2H). The patient was discharged without neurological deficits. The patient has had no rebleeding events for half a year.

Case 3

**Figure 3:**
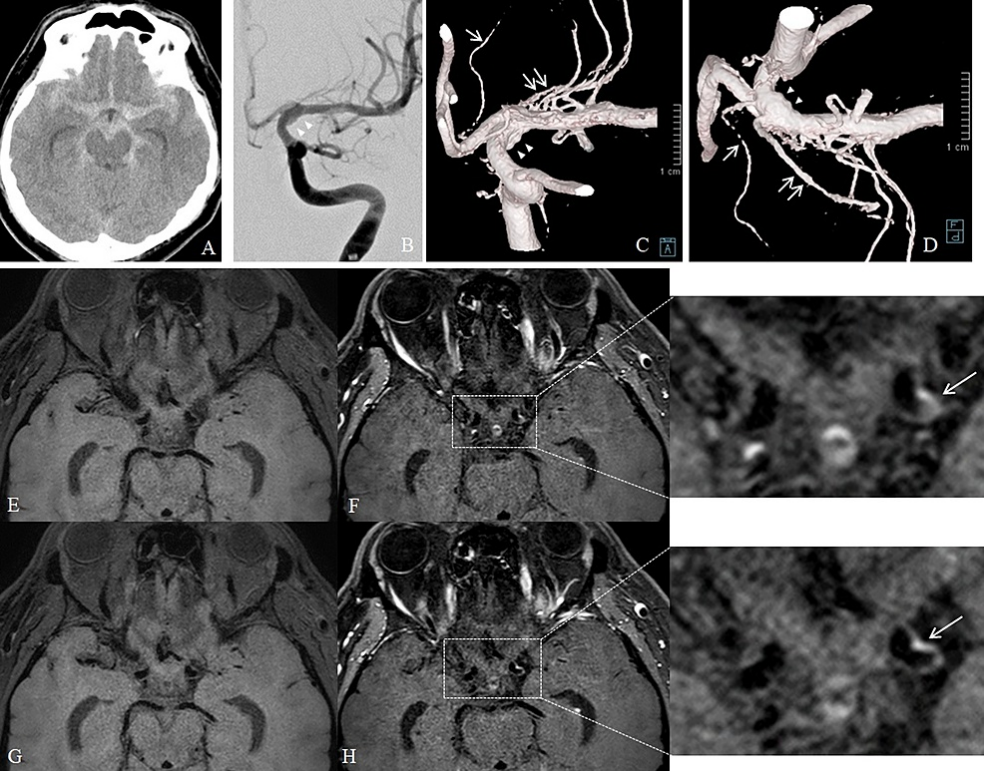
Case 3 (A) Computed tomography shows diffuse subarachnoid hemorrhage. (B-C) Digital subtraction angiography shows irregular dilatation (arrowheads) of the left internal carotid artery, which involves the posterior communicating artery (single arrow) and anterior choroidal artery (two arrows). (E-H) Axial views on magnetic resonance vessel wall imagings, (E, G) pre-contrast and (F, H) post-contrast administration, show enhancement of the dilatation wall (arrow).

A 38-year-old man presented with first sudden-onset headache. Two days later, he was transferred to our hospital because of second severe headache. CT showed diffuse SAH (Figure 3A). DSA demonstrated irregular dilatation of the lateral-posterior portion of the left ICA, which involved the posterior communicating artery and AChA (Figures 3B-3D). Next day, CE-VWI showed enhancement of the dilatation wall (Figures 3E-3H). The irregular dilatation was deemed to be dissection of the ICA or a blood blister-like aneurysm (BBA). The dilatation of ICA would have been difficult to manage using deconstructive treatment of parent artery occlusion with bypass surgery, because it was necessary to preserve posterior communicating artery and AChA. Therefore, endovascular treatment using a low-profile visualized intraluminal support stent without coils was performed. On the day of the procedure, dual antiplatelet agents were given orally. During the procedure, low-molecular-weight heparin was administered. A single stent was placed from the MCA to ICA near side of the origin of the ophthalmic artery. Four days later, diffusion-weighted MR images showed multiple small, high-intensity lesions on the left cerebral hemisphere. The patient was discharged without any neurological deficits. One year later, follow-up DSA showed that the irregular dilatation of the ICA had shrunk, with no evidence of recurrence.The patient has had no rebleeding events for one and a half years. This case with an intracranial stent was approved by our institutional review board as an experimental case (number: 3Komaki2787).

## Discussion

Wall enhancement of ruptured cerebral aneurysms on CE-VWI was first reported by Matouk in 2013 [4]. In five patients with aneurysmal SAH, including three patients with multiple aneurysms, all ruptured aneurysms showed wall enhancement, and none of the associated unruptured aneurysms showed enhancement. Nagahata et al. reported CE-VWI evaluations of 144 aneurysms, including 61 ruptured and 83 unruptured [5]. They classified wall enhancement into three groups: strong, faint, and no enhancement. Strong enhancement was defined as equal to that of the choroid plexus or venous plexus. The rates of “strong enhancement” were 73.8% and 4.8%, of “faint enhancement” were 24.6% and 13.3%, and of “no enhancement” were 1.6% and 81.9% in ruptured and unruptured aneurysms, respectively. When “faint enhancement” was defined as positive wall enhancement, aneurysmal wall enhancement demonstrated sensitivity of 98.4% and specificity of 81.9% for the detection of ruptured aneurysms. It is known that damage to the endothelial cell layer and inflammatory cell infiltration into the aneurysmal wall are greater in ruptured aneurysms than in unruptured aneurysms [7,8]. Mechanisms of strong enhancement of ruptured aneurysms are thought to be stagnation of the contrast material, endothelial damage, or inflammation within the aneurysmal wall [2,4-6].

Approximately 10%-20% of spontaneous SAH will have angiographically negative aneurysms, divided into two patterns of either perimesencephalic or diffuse SAH [9]. The etiology of the perimesencephalic SAH pattern is accepted to be venous hemorrhage. Nesvick et al. reported that initial DSA was negative in 103 (18.3%) of 563 patients with spontaneous SAH, and 36 of the 103 patients (6.4%) showed the diffuse SAH pattern [9]. In patients with the diffuse SAH pattern, repeated DSA detected a small ruptured aneurysm in three cases. Cerebral aneurysms of a maximum diameter less than 3 mm have been called as “small”, “very small”, or “micro” aneurysms [10-13]. Small aneurysms are a challenge for diagnosis and management because of their small size, fragile thin wall, and broad neck. The optimal management of small ruptured aneurysms remains controversial [10-13]. Chalouhi et al. reported a comparison of surgical clipping and endovascular treatment in patients with ruptured small aneurysms [10]. There were no significant differences in overall outcomes between the two treatments, although endovascular treatment had a significantly lower complication rate than open surgery.

The radiographic characteristics of ICA dissection are signs of regular or irregular fusiform dilatation, double lumen, intimal flap, and mural hematoma. The diagnosis of intracranial arterial dissection is challenging because the characteristic signs at times can be difficult to detect [14,15]. Meanwhile, intracranial BBAs can be located on the anterior wall of the ICA with a conical protrusion, which is sometimes characterized as a dissecting aneurysm or pseudoaneurysm [16,17]. Therefore, ICA dissection and BBAs have similar or overlapping pathogenesis. In cases where clip reconstruction or exclusion is not possible, trapping of the lesions is the most effective method to prevent rebleeding of ICA dissection or BBAs [14-17]. However, trapping of the ICA carries other untoward consequences, may require bypass, and is difficult if the lesion is located in the ICA segment involving the posterior communicating artery and AChA [18]. Recently, the utilities of a low-profile visualized intraluminal support stent for ICA dissection and BBAs have been reported [18-20]. Stent-only technique may be feasible and safe for some uncoilable intracranial aneurysms [19,20].

## Conclusions

Conventional vascular imaging detected scanty morphological changes in all cases reported here. However, CE-VWI information provided reliable confirmation of the lesions as bleeding points and provided confidence in surgical treatment. In cases 2 and 3, whether the lesions were true bleeding points could not be confirmed. Patients' good clinical courses over half a year without rebleeding suggest that the lesions were the bleeding points. However, there are several problems associated with CE-VWI for SAH. First, the time and stress of MRI in patients with untreated acute phase SAH may increase the risk of rebleeding. The logistics with patients who are intubated, critically ill or medically unstable may also be prohibitive. Second, interpretations of CE-VWI findings are occasionally difficult, especially several days after onset, because signals of SAH become high intensity on T1-weighted images. Third, many institutions are not equipped with 3T MRI facilities necessary for high-quality CE-VWI imaging.
